# Artificial Light at Night (ALAN): A Potential Anthropogenic Component for the COVID-19 and HCoVs Outbreak

**DOI:** 10.3389/fendo.2020.00622

**Published:** 2020-09-10

**Authors:** Zeeshan Ahmad Khan, Thangal Yumnamcha, Gopinath Mondal, Sijagurumayum Dharmajyoti Devi, Chongtham Rajiv, Rajendra Kumar Labala, Haobijam Sanjita Devi, Asamanja Chattoraj

**Affiliations:** ^1^Biological Rhythm Laboratory, Animal Resources Programme, Institute of Bioresources and Sustainable Development, Department of Biotechnology, Government of India, Imphal, India; ^2^Distributed Information Sub-centre, Institute of Bioresources and Sustainable Development, Department of Biotechnology, Government of India, Imphal, India; ^3^Biological Rhythm Laboratory, Department of Animal Science, Kazi Nazrul University, Asansol, India

**Keywords:** COVID-19, HCoVs, ALAN, bat, melatonin, sustainability

## Abstract

The origin of the coronavirus disease 2019 (COVID-19) pandemic is zoonotic. The circadian day–night is the rhythmic clue to organisms for their synchronized body functions. The “development for mankind” escalated the use of artificial light at night (ALAN). In this article, we tried to focus on the possible influence of this anthropogenic factor in human coronavirus (HCoV) outbreak. The relationship between the occurrences of coronavirus and the ascending curve of the night-light has also been delivered. The ALAN influences the physiology and behavior of bat, a known nocturnal natural reservoir of many *Coronaviridae*. The “threatened” and “endangered” status of the majority of bat species is mainly because of the destruction of their proper habit and habitat predominantly through artificial illumination. The stress exerted by ALAN leads to the impaired body functions, especially endocrine, immune, genomic integration, and overall rhythm features of different physiological variables and behaviors in nocturnal animals. Night-light disturbs “virus–host” synchronization and may lead to mutation in the genomic part of the virus and excessive virus shedding. We also proposed some future strategies to mitigate the repercussions of ALAN and for the protection of the living system in the earth as well.

**Graphical Abstract:**
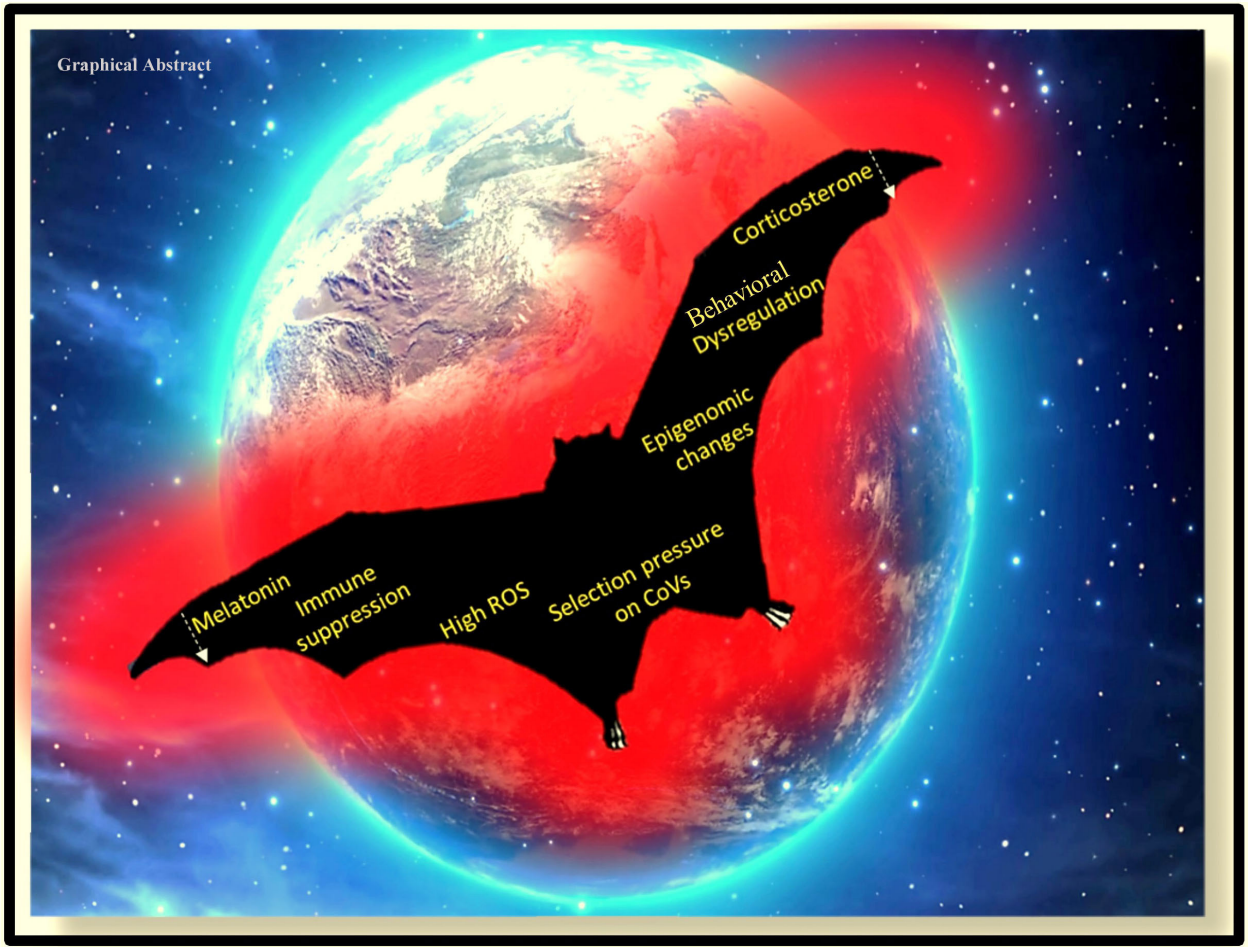
Impact of artificial light at night on the physiology and behavior of the nocturnal animal, the bat.

## Highlights

- Increase of anthropogenic Artificial Light at Night (ALAN) in due course of the “development for mankind” may be related to Coronavirus outbreak (HCoVs).
- Bats, nocturnal natural reservoir of many Coronaviridae, are heavily affected by ALAN due to the destruction of their proper habit and habitat.
- Most of the bat species are either “threatened” or “endangered” in IUCN list.
- Night-light might disturb “virus-host” synchronization by exerting selection pressure that may lead to the mutation in the genome of the virus and excessive viral shedding.
- Proposed strategies to mitigate the repercussions of ALAN and for the protection of our planet earth as well.

## Introduction

**Figure 1:**
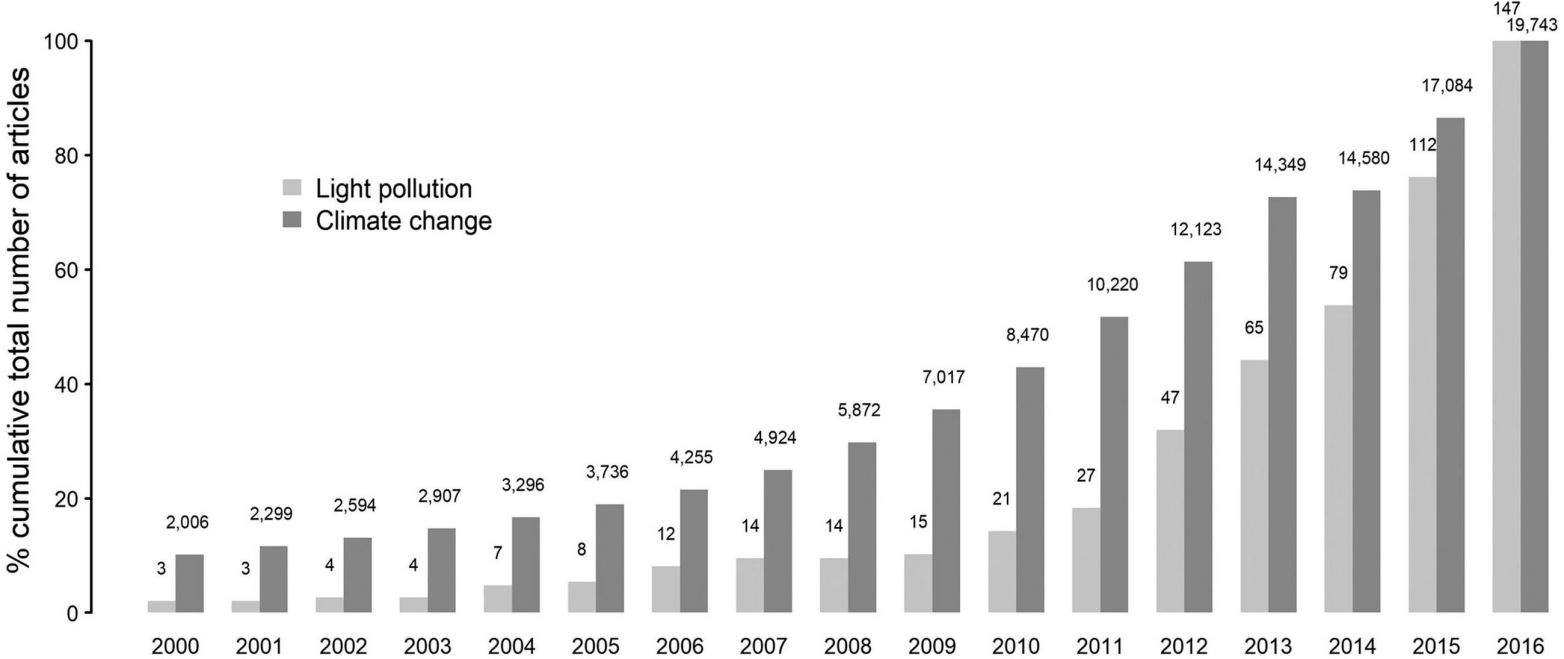
Representation of the trend in research outputs associated with light pollution and climate change since the year 2000. Adapted with permission from (1).

One of the most prevalent but least understood anthropogenic changes that impact living beings is the light pollution in the form of artificial light at night (ALAN). ALAN appears to be a massive threat to the growing human–environment conflicts, as it intervenes with all the three primary requirements (food, habitat, and health) for the sustainability of life in various animal species including humans (1–6). ALAN is one of the significant components of human-induced selection pressure, which has dramatically changed the trajectory, the rate of extinction, and speciation in this Anthropocene (7). In the case of nocturnal animals, such as bats and rodents, the threat imposed by the chief pollutants in the air, water, or soil seem to be less effective than ALAN due to its ubiquitous nature, level of influence, and diversity in biological response (1, 2). A number of studies in the last decade, by documenting the impact of ALAN on different ecological components and human health, indicated the severity and consequences of “light pollution” [Figure 1; (1)].

The detrimental physiological and behavioral effects resulting from the exposure to light at night are known (2, 8–10), but the influences of light pollution on the emergence and development of the infectious disease are yet to be elucidated. The appearance of new infectious diseases is increasing since the last decade and has posed a significant threat to public health worldwide. The origin of (60–80%) these emerging infectious diseases (EIDs) are reported to be from the wildlife (11). Among the EIDs, influenza, henipavirus, and coronavirus-related respiratory and neurological disorders have caused a severe threat to human health. Influenza viruses belong to the *Orthomyxoviridae* family, containing negative-single-stranded, segmented RNA genome (11). A majority of the seasonal influenza is associated with two types of influenza viruses, influenza A and influenza B (12). Influenza A in humans has originated from birds and swine (13). It is interesting to note that globally people get infected with influenza in the winter season due to the decrease in the ambient temperature and photoperiod (14). The introduction of influenza viruses in humans resulted in global pandemics (“Spanish flu” in 1918 and “swine flu” in 2009) followed by their continued circulation in human populations as seasonal flu. However, influenza B viruses have no known animal reservoir or flow within humans.

Coronaviruses (CoVs) represent a class of diverse genetic viruses found in a varied range of host species, including birds and mammals (15). CoVs are highly pathogenic single-strand RNA virus with a diameter of about 80–120 nm (16). They infect humans and other animal species, causing intestinal and respiratory infections. The number of confirmed cases of severe acute respiratory syndrome coronavirus-2/coronavirus disease 2019 (SARS-CoV-2/COVID-19) is much higher than that of severe acute respiratory syndrome coronavirus (SARS-CoV) in humans, due to more rapid transmission capability (17). Globally, the number of cases of COVID-19 are ascending steeply, overwhelming the governments, hospitals, and medical care, with 15,296,926 confirmed cases and 628,903 deaths and increasing as of 24 July 2020. CoVs can be divided into four types: α-coronavirus (α-COV), β-coronavirus (β-COV), γ-coronavirus (γ-COV), and δ-coronavirus (δ-COV) (18). Six CoVs were previously known to cause diseases in humans [SARS-CoV in 2002 and Middle East Respiratory Syndrome coronavirus (MERS-CoV) in 2012]. SARS-CoV-2 is the seventh member of the coronavirus [Table 1; (34)]. Recently, six novel CoVs have been reported from the bats in Myanmar (35). These six CoVs belong to the same family—the SARS-CoV-2—but distantly related (35). *In silico* sequence analysis gave the evidence of the emergence of SARS-CoV-2 from bats (36, 37). According to a few recent studies, weather plays a definitive role in spreading the infection of COVID-19 (38, 39), maintaining the characteristics of its ancestors, influenza. Despite understanding the mechanism of viral evolution and surveillance, new viruses continue to emerge and cause epidemics and pandemics around the world. The emergence of a novel viral strain is a result of genetic selection. The virus undergoes subtle genetic changes through mutation and major genetic changes through recombination. The main difficulties associated with the emergence of a novel viral strain are the scale of illness to human and other living organisms, although the processes that underlie the evolutionary dynamics of viruses and the timing and nature of the emergence of new virus strain remain unpredictable.

**Table 1:** Animal origins of HCoVs, Classification, natural reservoirs, outbreak species, year and country of outbreak.

**Human coronavirus**	**Classification of virus**	**Animal reservoirs (high prevalence)**	**Outbreak species**	**Country of origin/Year**	**References**
HCoV-OC43	Alpha-CoV	Mice, chickens, turkeys, swine, dogs, cats, rabbits, horses	Rodents	Russia/1890	(19–23)
HCoV-229E	Beta-CoV, lineage A	Mice, rats, chickens, turkeys, swine, dogs, cats, rabbits, horses	Bats	United Kingdom/1967	(19–21, 24)
SARS-CoV	Beta-CoV, lineage B	Masked palm civets, bats, rats, raccoon dogs, cats, swine	Bats	China/2002	(21, 25, 26)
HCoV-NL63	Alpha-CoV	Bats, mice, rats, swine	Bats	Netherlands/2004	(21, 27)
HCoV-HKU1	Beta-CoV, lineage A	Bats, mice, rats, swine	Rodents	China/2005	(26, 28)
MERS-CoV	Beta-CoV, lineage C	Cattle, chicken, bat, mice, alpacas, swine, dogs	Bats	Saudi Arabia/2012	(29–31)
SARS-CoV-2	Beta-CoV, lineage B	Bats, pangolins	Bats	China/2019	(32, 33)

Impacts of ALAN on animals range from constrained foraging, altered reproduction, and impaired communication (40–43) to a total swing in the trophic interactions (44–46). A recent study has reported that light pollution at night aids in the infectivity of the West Nile virus (WNV) in the house sparrow (*Passer domesticu*s) (47). Moreover, light pollution at night also altered the immune defenses of animals, including human by inhibiting the secretion of melatonin, a hormone which is known to enhance viral resistance and regulate immune response (48, 49). It has been established that the rate of secretion of melatonin is reduced following the exposure of light at night (50–53). The involvement of melatonin in immunomodulation is also reported in many studies on animals (54, 55). Exposure of Siberian hamster even to dim light leads to suppressing their immune response (56). It is evident that the acute respiratory disorder after coronavirus infection to human is mainly caused by an overstated immune response (cytokine storm) through inflammation and oxidation (57, 58). Melatonin, a well-known antioxidant and anti-inflammatory agent is protective to critical care patients and is known to act through reducing the permeability of vessels and anxiety and improving sleeping quality (58). Moreover, the existence of identical biosynthesizing machinery for melatonin in several organs in animals (59–66), a subcellular component like mitochondria (67), vouches for the importance of melatonin in cellular physiology.

Bats are known to harbor a wide variety of viruses ranging from coronavirus to ebolavirus to henipavirus (68), without showing any clinical symptoms of the diseases concerned. Their long life span might be the outcome of an intricate balance between the host immune system and virus infection (68). In this communication, we reviewed the relationship between the growing use of ALAN and the ALAN linked threats to bats, as they are the primary reservoirs of CoVs. The focus is also given to the influence of ALAN on the activities of the bats and the virus–host interaction. We tried to frame some future strategies for the prevention of this type of unpredictable zoonotic virus outbreak along with some possible treatments for ALAN-induced reservoirs and infected humans.

## Impact of Artificial Light at Night on the Living System—Emergence of the Idea in the Global Scenario

### Artificial Light at Night and Melatonin—The Physiological Messenger of Environmental Darkness

The circadian and seasonal variations in the animal physiology are directly or indirectly regulated by melatonin, which is further dependent on environmental photo-thermal conditions (61, 69). Light suppresses the synthesis and release of melatonin from the pineal gland and acts as the primary zeitgeber for synchronizing internal rhythms to the temporal change of the external light and dark cycle. Under natural light–dark conditions, melatonin biosynthesis in the pineal gland of most animals including human, bats, rodents, and fish reaches its peak at midnight (70–75). Depending on the species, the biological rhythm and melatonin secretion are controlled by various organs such as the hypothalamic suprachiasmatic nucleus (SCN) and the retina (in mammals) and the brain, pineal, and retina (in fish and amphibians); nonetheless, most animals follow conserved norepinephrine and adrenergic receptor pathways (73, 76–79). The duration of melatonin biosynthesis and secretion is the pivotal parameter for the day-length signaling, which is essential for the organization of the seasonal rhythms (80). Studies following the administration of physiological concentrations of melatonin at the proper time in pinealectomized hamsters and sheep demonstrated the dose- and time-dependent roles of melatonin in the transmission of day-length signaling in animals (80). Further, the annual breeding cycle has also been found desynchronized in pinealectomized sheep (81). In ruminants, melatonin consumption (through food) in summer (before the onset of darkness) can mimic the early onset of seasonal reproductive function, which includes winter coat growth and the suppression of secretion of prolactin, characteristics of reproductive behavior of winter photoperiod (82). Studies demonstrate the importance of daily and seasonal melatonin rhythmicity profile in different animals. However, owing to ALAN, disruption in the diurnal and seasonal variations in the physiology and behavior has been reported in several animal species including humans, bats, and fish (53, 83–85). Light pollution is a global problem, a fact supported by the resolution of the American Medical Association (AMA), declaring that light at night is a source of environmental pollution as it disrupts daily rhythms and suppresses nocturnal melatonin biosynthesis (83, 86). The amount of light required to suppress the melatonin biosynthesis and secretion is dependent on both species and photoperiod (80). For instance, some laboratory-raised animals require less light than the same species raised in the wild (80). In humans, it is identified that the light spectra of between 440 and 482 nm are responsible for the peak melatonin suppression and pupillary constriction (87, 88). Furthermore, it is evident that polychromatic light enriched with short wavelength results in the suppression of melatonin (80, 89, 90). Various cross-sectional studies have pointed out that ordinary domestic light can elicit 50% of the maximum response by phase resetting the diurnal rhythm of melatonin, cortisol, and body temperature (91, 92). Animals exposed to prolonged day-light during summer are found to be partially resistant to the melatonin suppression during night-light (93). Additionally, Morita et al. found that differences in the phases of the diurnal melatonin rhythm depend on the level and pattern of exposure to light (94). The “hypothalamic light perception” may induce the suppression in the melatonin biosynthesis through ALAN even in blind patients (95). The hypothalamic light perception was studied in animals in which the retinohypothalamic projection was intact; but the primary and accessory optic tracts were surgically removed (80, 95). Cumulatively, these results demonstrate the inhibitory effect of ALAN on the diurnal and seasonal rhythmicity of animals. Moreover, it is also pertinent that melatonin suppression by ALAN is dependent upon the level of exposure to bright light during the daytime and season (96). A comprehensive study on the seasonal or diurnal melatonin rhythm and ALAN on the bats is lacking. Nevertheless, there are several studies that demonstrate that the concentration of melatonin in bats at night ranges from 60 to 500 pg/ml while human nocturnal melatonin level ranges from 11 to 83 pg/ml (74, 97, 98). It is evident that the melatonin level in bats is higher than in humans; therefore, it should have an extremely significant contribution in maintaining bat physiology. It is also found that *Rhinolophus* bats are nocturnal and remain hidden in dark places and thereby less exposed to day-light. This behavior makes them more susceptible to melatonin suppression by ALAN than other animals that are exposed to light in the daytime (97). This suppression of melatonin secretion may be extremely high, which negatively influences the physiology of bats and possibly also on the virus residing in them.

### Emergence and Evolution of Artificial Light at Night—A Chronological Country-Wise Scenario

In Russia, around 1890, when HCoV-OC43 crossed the species barrier to infect humans, a pandemic of respiratory infection was observed (23). The second episode of HCoVs named HCoV-229E originated in the 1960s in the United Kingdom (24). Genetically, HCoV-229E is closely related to bat alpha-CoVs; and between alpha-CoVs and HCoV-229E, there exists an alpaca-CoV (32). The direct transmission of previously known CoVs from bats to humans has also been reported (99). Ironically, Russia and the United Kingdom were pioneers in gas street lighting. In 1835, the company for gas lighting was established in St. Petersburg. By 1860s, most of the central streets and buildings of the capital were well-illuminated with gas street lighting. More gas works were functional in the 1870s, and by 1882, Moscow was shining with 10,000 gas lamps. In 1888, almost 210 gas works were operating in Russia, including 30 for lighting cities, 157 for factories, and 23 for railway stations (100, 101). Meanwhile, the United Kingdom became the first country in the world to be lighted by an electric bulb after the development of 16-W lightbulb by Swan/Edison in 1880 [Table 2; (123)]. The next significant advancement in artificial lighting was the invention of the sodium-vapor lamp by Compton and light-emitting diode (LED) by Oleg, in 1920 and 1927, respectively [Table 2; (124, 125)]. The night lighting quickly advanced with halogen and high-pressure sodium-vapor (HPS) lamp (126, 127), both of which were capable of emitting uninterrupted high-intensity light and became a powerful tool for street lighting. Till that time, the artificial lighting was minimal; most countries did not have electricity. Similarly, the HCoV outbreaks were limited. With massive development in electricity production (hydrothermal and nuclear), artificial light reached the untouched regions. Swiftly, artificial lighting tools became ubiquitous; after the USA and Europe, the Asian continent started to use them for lighting at night. The invention of LED, organic LED (OLED), and liquid crystal display (LCD) screens has further contributed to the artificial lighting.

**Table 2:** Timeline of the development of Anthropogenic Light sources on Earth.

**Year**	**Artificial lighting technology**	**Inverter/Country**	**References**
1780	Argand lamp	Aime Argand/Geneva	(102)
1792	Gas lighting	William Murdoch/England	(103)
1800-1809	Arc Lamp	Humphry Davy/England	(104)
1856	Geissler Tube	Heinrich Geissler/Saxe-Meiningen	(105)
1867	Fluorescence lamp	A. E. Becquerel/Paris	(106)
1875	Incandescent light bulb	Henry Woodward/Canada	(107)
1880	Long lasting filament	Thomas Edison/USA	(108)
1894	Gas discharge lamp	D. McFarlan Moore/USA	(109)
1901	Mercury-vapor lamp	Peter Cooper Hewitt/USA	(110)
1904	Tungsten filament	Alexander Just and Franjo Hanaman/Hungry	(111)
1910	Neon lighting	Georges Claude/France	(112)
1913	Inert gas in bulb	Irving Langmuir/USA	(113)
1920	Sodium vapor lamp	Arthur H. Compton/USA	(114)
1927	Light-emitting diode	Oleg Losev/Russia	(115)
1953	Halogen light bulb	Elmer Fridrich/USA	(116)
1962	Red light-emitting diode	Nick Holonyak Jr./USA	(117)
1963	High-pressure sodium vapor lamp	Kurt Schmidt/USA	(118)
1987	Organic light-emitting diode (OLED)	Ching W. Tang and Steven Van Slyke/USA	(119)
1995	Blue LED	Shuji Nakamura/Japan	(120)
2008	LED lighting system with helical fiber filament	G. R. Hulse/USA	(121)
2019	LED filament chips	T. Jiang/Japan	(122)

**Figure 2:**
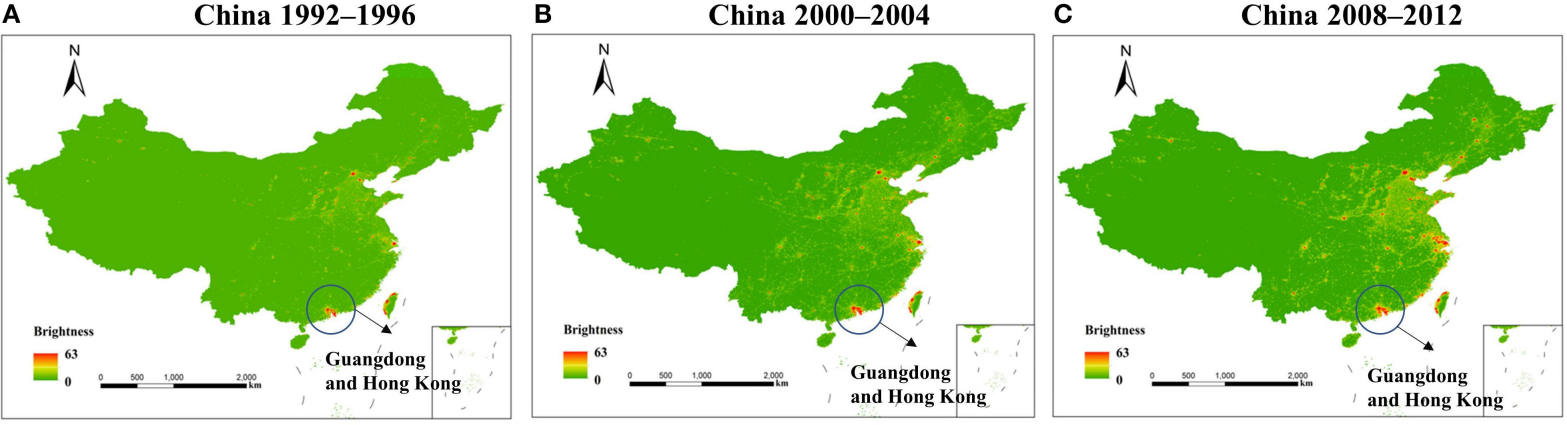
Pixel brightness (calibrated digital number) mean value 1992–1996 **(A)**, 2000–2004 **(B)**, and 2008–2012 **(C)**; Guangdong and Hong Kong were encircled as the two centers of human coronavirus (HCoV) origin. The figure is used under the terms of the Creative Commons attribution license (128).

**Figure 3:**
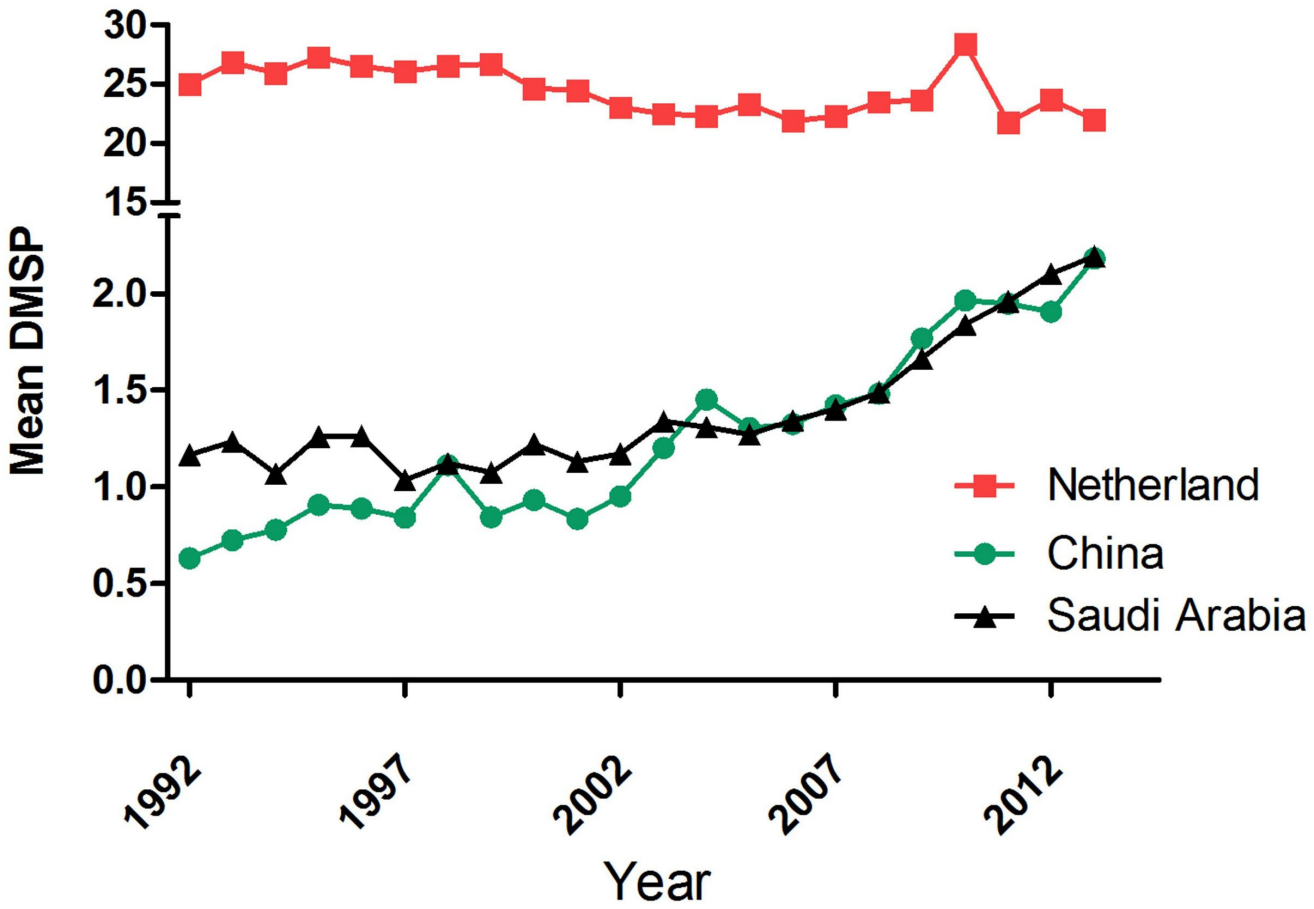
Annual mean the defense meteorological program (DMSP) level in (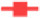) the Netherlands, (
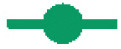
) China, and (
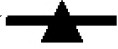
) Saudi Arabia from 1992 to 2013. Data were obtained from https://www.lightpollutionmap.info.

In the last two decades, five incidences of the HCoV outbreaks have emerged, which is 250% more than the previous 110 years. Similarly, the use of artificial light has increased manifold in the past two decades than in the last century (1). With the beginning of the twenty-first century, another pandemic SARS-CoV surfaced in Guangdong Province of southern China. The artificial lighting was very pronounced in Guangdong Province as compared with the other parts of China (Figure 2). Soon after SARS-CoV, another outbreak was observed in 2004 in the Netherlands, named HCoV-NL63. The ALAN was high in the Netherlands starting on the late 20th century (Figure 3). Another incidence of HCoVs, the HCoV-HKU1, took place in the next year of HCoV-NL63 in Hong Kong, which is also a massively lit region of China. Subsequently, the world has seen the outbreak of MERS-CoV and SARS-CoV-2 in Saudi Arabia and China, respectively (Table 1). These two were rapidly developing countries, with quick urbanization and economic development; it was inevitable to control light pollution.

**Figure 4:**
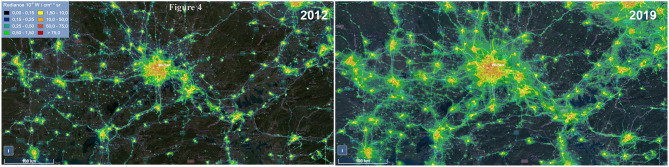
Image showing the change in the level of artificial light at night (ALAN) in Wuhan from 2012 to 2019. Data were obtained from https://www.lightpollutionmap.info.

It may be noted that diurnal animals such as chickens, turkeys, swine, dogs, cats, rabbits, horses, and cattle are also the reservoirs of the coronavirus (25, 129). However, interestingly, all the seven zoonotic transfers occurred from the nocturnal animals, mostly bats (Table 1). Out of the seven outbreaks, three major events of HCoV outbreak happened in China. This 42% outbreak in China is probably related to the fact that these animals are kept in captivity in markets that are usually well-lit at night. The present COVID-19 outbreak is reported to be from Wuhan, China, which also witnessed a massive increase in the ALAN in the past decade (Figure 4). Like ALAN, the population density of human in these countries has also increased over time. It can be hypothesized that increased human density and activities, especially, deforestation may also be potential factors for HCoV outbreak, as they can also exert selection pressure on the virus residing in the bats. Considering the significant impact of ALAN on bats, it may be assumed that the population growth supplements the negative effect of ALAN on bats. The evidence from historical and technological comparative assessment might be circumstantial but is good enough to provide the basis of the belief that artificial light plays a significant role in the outbreak of HCoVs.

### The Bats as the Natural Reservoirs of Human Coronaviruses

Bats are reservoirs (asymptomatic to the disease) of many viruses, including CoVs that cause severe diseases in humans and animals. The EIDs, mostly caused by the pathogen associated with wildlife species, are a critical threat to human and animal health (130–134). Since the past few decades, the fatal epidemics like acquired immunodeficiency syndrome (AIDS), SARS, filoviruses (e.g., Ebola and Marburg viruses), swine acute diarrhea syndrome (SADS), porcine epidemic diarrhea (PED), and influenza are viral diseases that originated from wildlife species (130), mostly from bats (134, 135). Among many bat-borne RNA viruses, two families of positive-sense single-stranded RNA viruses, namely *Astroviridae* and *Coronaviridae*, are important because of their high transmissibility (136). It is also reported that 100 bat species are found to be the reservoir of viruses causing the disease in animals in the Americas, Africa, Europe, Australia, and Asia (137, 138). The person-to-person transmissibility of CoVs and severe diseases associated with them have enhanced the urgency to study them, as revealed by SARS-CoV (139), MERS (140), and COVID-19 (141).

### The Effects of Artificial Light at Night on the Bats

Existing literature on the effects of ALAN on bats necessitates the integration of information on their different behaviors to emphasize the possibilities of the emergence of several fatal viruses.

#### Behavioral Pattern

Anthropogenic encroachment of natural habitats of animals through deforestation, habitat fragmentation, urbanization (over occupying agricultural land), and bushmeat consumption are considered as the primary drivers that are promoting the interspecies transmission of pathogens from wildlife reservoirs to humans (142–145). Global urbanization and human development by anthropogenic interventions led to a dramatic increase in both the extent and intensity of artificial lighting throughout the twentieth and twenty-first centuries [Table 2; (146, 147)]. The use of ALAN is increasing annually by 6% worldwide (148) and causes nocturnal sky brightness by 20% (9, 149, 150). Light pollution affects every inhabitant of the ecosystem. The habitats of bats are affected either through direct loss or disturbance from artificial light or human activities. Further, connectivity to roosts from foraging areas is fundamental for the survival of many bat populations and is also affected with light at night and so-called development, meant for the human activity only (151). It is evident that the light–dark cycle maintains the daily pattern of activity and behavior in bats (152). The sunset influences the timing of the nightly emergence of bats from the roost; on the other hand, moonlight (153) and the length of night affect the foraging activity and overall behavior of bats (154, 155). The significant impact of ALAN upon bat behaviors, including foraging and commuting, emergence, roosting, breeding, hibernation, and abundance have been recorded (156), but detailed information is not yet available and thereby warrants further investigation.

#### Commuting Behavior of Bats

The ALAN splits the bats' commuting routes or flyways between roost and foraging areas, causes avoidance behavior, and thereby, fragments the network of flyways. Many species avoid their flyways, which are illuminated with HPS and LED (157–161). As a result, bats are forced to use alternate routes to reach their foraging grounds. This alteration increases energy expenditure due to the enhancement in flight time and also the risk of predation (158). If the alternate route is not available, bat colonies are found to be isolated from their foraging areas and abandon their roost (159). Some light-tolerant bat species pay attention to the streetlights for feeding as the higher number of insects (particularly moths) get attracted toward the lamplight (162). This feeding and foraging behavior increases mortality risk due to collision with the vehicles and predation risk for the juvenile (due to their slow and less agile flight) (163), as also found in many birds (41). Light-sensitive bat species lose their foraging fields due to quick passage in the lit area (164). Further, composition and abundance of prey (insect) for bats also change in the illuminated regions (165).

#### Emergence, Roosting, and Breeding Behavior

The dusk period is the time for the onset of the emergence of bats due to the availability of insects in the foraging areas (166). Artificial light delays or hampers the time and duration of the emergence of the bats and, thereby, reduces the quality of foraging time and negatively affects the fitness of bats (167). Repeatedly alternating exit/entrance due to ALAN forced bats to abandon the roost and become entombed in the worst cases (168).

External and internal lighting in and around the bat roost causes reduced fitness and hinders juvenile growth rates. As a result, it makes the animals immunodeficient and susceptible to the different pathogens at a tender age and also increases the threat of predation (167). The changes in the internal physiology of the bats may also influence their ability to be the reservoir for different viruses. By virtue, viruses may mutate to find a different host for their sustainability.

#### Hibernation

The hibernation is a period when bats allow their body temperature to decrease below the active homeothermic level to conserve energy on a seasonal basis in response to the changes in the environmental temperature and food supply (169). The suitable microclimatic conditions allow efficient energy budgeting in bats during this hibernation to survive in winter (170). The stimulation from the artificial lighting during the hibernation of bats results in the significant energy expenditure, lowering fitness and thereby reducing the chance of survivability in the winter and subsequent spring (171). Moreover, artificial light may disrupt circadian rhythms during the hibernation in bats (171); a similar phenomenon of rhythm desynchronization is found even in non-hibernating animals (2, 9).

The anthropogenic disturbances by ALAN cause chronic stress (elevated levels of plasma glucocorticoid hormones) and disruption of homeostasis, which may be due to the desynchronization of the circadian rhythm in bats (172, 173). Stress-induced immunosuppression may increase the susceptibility of the bats to acquire and shed viruses (174). Even hot climatic conditions or long periods of high temperature stimulate rapid amplification and increase the transmission risk of WNV to vertebrates from wild birds (175).

As an obvious outcome of the above-cited studies, the bats have been logically designated as “threatened/endangered” by the International Union for Conservation of Nature (IUCN) (http://www.batcon.org/why-bats/bats-are/bats-are-threatened). The IUCN listed 24 bat species as critically endangered, 53 others are endangered, and 104 bat species are considered vulnerable. Almost one third of the 1,296 bat species that have been assessed by the IUCN are either threatened or data deficient, indicating the need for more attention for their conservation. This status of the bats might explain the reasons for the viral shedding, the mutation, and the adaptation of these microorganisms to new hosts, and ALAN is one of the crucial factors for that.

#### Artificial Light at Night and Virus–Host Interaction

The appearance and subsequent circulation of influenza occur along the latitudinal belts and coincide with the changes in the photoperiod (176). This seasonal influenza is synchronized by solar elevation, day length, and solar isolation (176). Influenza mortality in elderly people is probably due to limited sun exposure (176). These studies link the viral infection with the photoperiod; however, there is a lack of information on the effect of ALAN on host–virus interaction, viral evolution, or outbreak.

It is well-accepted that rapid mutation and genetic recombination results in the emergence of novel HCoVs (177, 178). Mutation in the spike (S) protein of the virus might increase the affinity with human angiotensin-converting enzyme 2 (ACE2) receptor (129). Apart from the mechanism mentioned above, the evolution of HCoVs might also be driven by the host-associated selection pressure. However, less is known about the selection pressure exerted by the host on their reservoir community. The reservoir of the CoVs is large and mostly include bat species, and it can be easily hypothesized that CoVs are well-adapted with the anatomy and physiology of bats (179). Furthermore, asymptomatic or minimal disease symptoms were observed when bats were infected with CoVs (135), indicating that bats and HCoVs are mutually adapted.

This interaction of bats and HCoVs may seem beyond the reach for most of us, but they are not beyond our influence. Humans have indirectly disturbed the physiology and behavior of the bats (156) with inconsiderate industrialization and urbanization. Increase in the amount of ALAN has a widespread effect on wild animals, including fish, marine turtles, birds, and nocturnal animals, including bats (8, 9, 40, 180, 181). Impacts of ALAN on nocturnal animals range from constrained foraging, altered reproduction, and impaired communication to a complete shift in trophic interactions and alteration in community structure (40, 45, 182). In plants, it has been reported that appropriate lighting environment is essential for the development of a comprehensive resistance system in various plant–pathogen interactions, including viruses (183). Moreover, artificial light has enhanced the development of the disease in *Nicotiana tabacum* after inoculation with cucumber mosaic virus (CMV) (184). Similarly, in animals, seminal research pointed out that the influence of light pollution can extend the infectious-to-vector window for WNV by 41% in the house sparrow (*Passer domesticus*), an urban-dwelling avian reservoir host of WNV. This indicates that light pollution can directly aid in the virus transmission to humans (47). Light pollution at night causes the sparrow to produce more corticosterone, which alters the regulation of avian physiology via stress-response pathway (47). Similarly, ALAN-induced stress can modulate the pro-inflammatory response in bats, which can efficiently reduce the pathology triggered by CoVs, implying a direct connection between ALAN and bat–HCoV interaction (185). The experimental data on the prolonged exposure of ALAN on zebrafish also indicated a similar enhanced inflammatory response by TNF-α and NF-κβ pathways (9). Moreover, light pollution at night also altered the immune defenses by inhibiting the secretion of melatonin, a chronobiotic hormone that enhances viral resistance, regulates immune response, maintains the level of reactive oxygen species (ROS), and acts as a mediator between the environment and epigenome (83, 186, 187). The high degree of ROS could suppress the replication of CoV and could alter proofreading activity of exoribonuclease (179). Cumulatively, ALAN can change the level of corticosterone, melatonin, viral resistance, immune response, and epigenome along with the high level of ROS. These factors are more than adequate to disturb the natural balance between bats and CoVs and exert a selection pressure on the virus to find a novel host through the mutation in the genetic structure of virus. Therefore, ALAN should be considered as a potential factor that is causing the emergence of the present COVID-19 and previous CoVs. The emergence of five novel CoVs in two ALAN-driven decades should not be considered as a matter of chance or a laboratory construct.

### Efficacy of Melatonin in the Reduction of Oxidative Stress and Immune Defense

The antioxidant property of melatonin is mediated by its inherent free radical scavenger activity, up-regulating anti-oxidative enzymes, and down-regulating pro-oxidative enzymes (e.g., nitric oxide synthase) (188, 189). Along with antioxidant property, melatonin has high bioavailability as it can penetrate the blood–brain barrier and placenta (190, 191). Melatonin reduces molecules or particles, which cause oxidative stress along with an increase in anti-oxidative enzymes, such as superoxide dismutase, glutathione peroxidase, and catalase activity (191–194). Viral infection causes oxidative stress by elevating levels of ROS and/or nitrogen species (RNS) (195). Similarly, the high expression of oxidative stress-sensitive gene Group IID secretory phospholipase A2 (PLA2G2D) reduces anti-viral immunity of the organisms (97). Oxidative stress reduces the number and activity of protective immune cells by stimulating immunosuppressive mechanisms, thus producing a pro-inflammatory response (196). Like the neuroendocrine system, the immune system has its circadian rhythm. The production of granulocyte, macrophage, and its phagocytic activity correlates with the nocturnal peak of melatonin (197, 198). Any change in the circadian system can desynchronize the immune system. Melatonin also regulates the immune system and enhances the immune response by improving proliferation and maturation of natural killer cells, T and B lymphocytes, granulocytes, and monocytes in both bone marrow and other tissues (199). Recently, melatonin is considered as a potential adjuvant for improving clinical outcomes of COVID-19 patients (97, 200, 201), though a detailed study regarding the efficacy of this indoleamine in host–virus interaction in both bats and humans is warranted (202, 203).

## The Future Strategies to Mitigate the Repercussions of Artificial Light at Night and to Minimize Virus Outbreak From the Bats

Multiple cross-sectional studies have proved that there is no substitute for natural darkness, and any change in the lighting might have severe implications as observed in several animals (9, 204, 205). On the basis of the research, we want to put forward some strategies to minimize the effect of ALAN on virus outbreak from the bats, like HCoVs.

### Evasion

The easiest and effective means to reduce the impact of lighting on bats is to define the bat zones and avoid illuminating them. The use of part-night lighting (PNL: switch off the lights between midnight and 05:30 a.m.) can be imposed in the illuminated bat zones. The PNL will help in limiting the adverse effects of light on bats and other nocturnal animals (206). If it is unavoidable to use the lights in the bat zones, a physical barrier should be built to reduce the area of illumination. In the newly developing sites, with a little bit of research, light exclusion zones (dark regions) can be created to allow movement of bats.

The banning of the bats as food item will reduce the captivity stress and exposure of light on them in the market. This strategy will also help to maintain the bat population.

### Use of Artificial Intelligence

Artificial intelligence (AI) coupled with high-resolution infrared cameras may be utilized to develop an artificial system that can distinguish between the human and wild animals at night. Lin et al. have developed a framework consisting of optical deep learning methods, through neural networks using multiple layers of diffractive surfaces programmed to execute subjective task that resulted in the statistical learning of the network through the computer (207). This method can be implemented to create a camera system that can capture and analyze any fast-moving object or an animal, such as the bats, and can classify them based on machine learning and deep neural network. The camera system should be off at all the time unless it detects a human, or the program will turn off the lights of the zone in which bats are flying. This strategy can be modified as per the need of the area to be monitored.

### Variable Lighting Regimes/Planned Positioning of Artificial Lights

As avoidance is not possible in all the scenarios and AI can be expensive, alternatively, a careful study should be conducted to develop variable lighting regimes (VLRs), which will be compatible for both human being and the wildlife. It has been suggested that tree cover can help in mitigating the adverse effects of ALAN on the bats by shielding the light (208). A careful study of tree cover and other shades before installing artificial lighting might help to shield the bats from ALAN. Moreover, adding trees in already lit areas will help in reducing the repercussions of ALAN on the bats. Horizontal or upward emission contributes substantially to light pollution by generating skyglow; this can be significantly reduced by using directional lighting (209). As poorly designed luminaires cause most of the light pollution and skyglow, effective luminaire design, installation of shielding fixtures, and correct column height can reduce the skyglow. Hedgerows, the vegetation canopies, can also be used to decrease light exposure, because many bat species use linear features as traveling routes (210). Even though developing a tree fence is very promising and might help the environment in multiple ways, including the reduction of the effect of ALAN, the negative impact of ALAN on the plants should also be considered. Therefore, utmost care should be given in the selection of plant species for developing the tree canopy.

### Changing the Type and the Intensity of the Artificial Light

Several studies have shown that bats are equally active in red light and darkness (211). Therefore, careful selection of lighting wavelength is paramount to reduce the stress of ALAN on the bats and other nocturnal animals. The red light should be used in places where it is unavoidable to limit the timing of illumination. Similarly, some bats and insects species thrive better in low-intensity lighting (212). So lower-intensity lights can be utilized to mitigate the negative impact of ALAN on bats. These two strategies will inevitably be a compromise among human necessities. However, these minor changes do not appear to be a bad deal if they can help in avoiding the outbreak of pandemics and protect species from getting extinct.

### The Use of Melatonin Spray as Reversal Therapy for the Treatment of Artificial Light at Night-Exposed Animals

Exposure of light on the bats can decrease their level of melatonin (74). Melatonin has potent antioxidant activity and anti-inflammatory activity, maintains biological rhythm, and protects against lipid peroxidation (213, 214); thereby, the reduction in the level of melatonin causes severe consequences on animal physiology. Recently, researchers have developed melatonin-loaded nano-capsule, spray-dried powders, and hydrogels to improve their stability even in the aqueous solution (215, 216). These nano-capsules can be sprayed on the animals that are already exposed to ALAN. This melatonin spray will decrease the inflammation in the bats and might also help in minimizing the level of selection pressure on the HCoVs residing in the bats.

### Exploratory Research

Previous studies demonstrated that CoV genomes display a high degree of plasticity in terms of gene content and recombination (32). Furthermore, relatively large CoV genome increases the probabilities for adaptive mutations, making it easier for the viral spike protein to exploit cell surface receptors of other species for virus attachment and entry (32, 33, 217). Therefore, exploratory research is warranted to understand the factors determining the emergence and evolution of the novel pathogens like COVID-19. Further, emphasis should also be given to the factors (including anthropogenic stress like light pollution) that increase the rate of selection pressure, transmission, and infection. It is of note that the deadly HCoVs come mainly from their nocturnal reservoir host. More research is required to find out the other host of CoVs in the wild and potential factors that are causing the evolution and origin of HCoVs.

### Transdisciplinary Approach

Multiple disciplines should collaborate to study the origin and evolution of HCoVs and to develop rules and guidelines to minimize such pandemic outbreaks. Scientists, policymakers, and engineers need to work together to implement strategies to reduce the impact of artificial light on bats. Finally, it is imperative to expand awareness of light pollution and its ecological impacts.

## Conclusion

The present review is a meaningful attempt to correlate the development of ALAN and emergence of HCoVs. The influence of ALAN on bats including inappropriate foraging and commuting, untimely emergence, roosting, breeding, and impaired physiology have put them under the threatened status in the IUCN list. ALAN-induced behavioral and physiological stress might have exerted an immense amount of selection pressure on the diverse form of CoVs residing in the bats. Except for the study on WNV by Kernbach et al. (47), most evidence of the impact of ALAN and virus infectivity is correlational; therefore, more studies are required to confirm the role and potential of ALAN in virus outbreaks from wildlife. Moreover, the effect of ALAN on farmed and domesticated animals is mostly unknown. The future research should be focused on these animals to prevent any outbreak of anonymous zoonotic transmission, influenced by ALAN. Given the fact that bats carry a variety of viruses with the capability to infect human and other organisms, it is essential to monitor wild animal species for any novel zoonosis. A global surveillance network involving veterinarians and animal biologists is urgently needed to monitor, and possibly to predict, potential sources for the emergence of other highly pathogenic CoVs. Besides, as the entire human population goes under lockdown, there is a surge in the use of ALAN; the classes, meetings, and cinema are all online. Presently, we are exposed to ALAN more than ever in the history of mankind and also in the companionship of arrhythmic lifestyle. Strict measures should be taken to minimize this exposure. Otherwise, we will be facing a huge group of human beings with lifestyle disorders.

Scientists are furnishing data since the last decades about the detrimental effect of ALAN and are recommending various strategies to reduce the effect (https://www.anses.fr/en/content/leds-anses%E2%80%99s-recommendations-limiting-exposure-blue-light). The earth is rhythmic in both circadian and circannual patterns, the human physiology is synchronized with the “natural light,” and any desynchronization in these processes may lead to an unmatched pandemic, to which we are fighting.

## Author's Note

This paper reviewed the potentiality of the artificial light at night (ALAN) in the outbreak of the HCoVs.

## Author Contributions

ZK, TY, GM, SD, CR, RL, and HS drafted the original of the respective subsections. ZK and CR prepared the figures. ZK and GM reviewed the manuscript. AC developed the concept with thorough literature searching and discussion in the group, made the outlines, analyzed the subsections through review, and critically edited the paper. All authors contributed to the article and approved the submitted version.

## Conflict of Interest

The authors declare that the research was conducted in the absence of any commercial or financial relationships that could be construed as a potential conflict of interest.
